# Hidden totipotency in naïve human pluripotent stem cell cultures

**DOI:** 10.1093/lifemedi/lnac024

**Published:** 2022-07-21

**Authors:** Daniel A Schmitz, Jun Wu

**Affiliations:** Department of Molecular biology, University of Texas Southwestern Medical Center, Dallas, TX 75390, USA; Department of Molecular biology, University of Texas Southwestern Medical Center, Dallas, TX 75390, USA; Hamon Center for Regenerative Science and Medicine, University of Texas Southwestern Medical Center, Dallas, TX 75390, USA; Cecil H. and Ida Green Center for Reproductive Biology Sciences, University of Texas Southwestern Medical Center, Dallas, TX 75390, USA

Capturing totipotency in a dish has the potential to revolutionize basic science and translational medicine. Human totipotent-like cells have now been found within cultures of naïve human pluripotent stem cells and are able to give rise to both embryonic and extraembryonic tissues.

The understanding of human early embryogenesis is key to developing treatments for human developmental disease and infertility, but it has been hampered by ethical, legal, and technical limitations involving research using actual human embryos. Because of this, researchers have developed various *in vitro* models of early human embryogenesis to act as accessible proxies for human embryos.

The workhorse of the early embryo modeling field has been the pluripotent stem cell (PSC). These cells have the remarkable ability to give rise to any cell in the adult body and can ­self-renew indefinitely in culture. In the context of embryogenesis, PSCs act as the *in vitro* representation of the pluripotent epiblast which gives rise to the embryo proper but not extraembryonic tissues. Thus, in general, PSCs lack the developmental capacity to give rise to extraembryonic tissues. Because of this limitation, a great effort has been made in recent years to generate *in vitro* cell lines that have an even greater developmental capacity than that of PSCs: totipotent stem cells.

By the strictest definition, totipotency refers to the ability of a cell to give rise to an entire embryo including extraembryonic tissues. Totipotent cells have the highest developmental potential of any cell, and in mouse embryos totipotency lasts for ~1.5 days where only the one- and two-cell stage blastomeres are truly totipotent. The establishment of totipotent stem cells *in vitro* has the potential to revolutionize early embryo research, reproductive biology and regenerative medicine, and would allow for the generation of any cell, tissue, or, potentially, organism from a single cell type. However, the identification and stabilization of stem cells with totipotent characteristics *in vitro* proved to be exceedingly difficult, as it took nearly 30 years after the first derivation of PSCs to generate mammalian totipotent-like cells in culture.

A decade ago, Macfarlan et al. published a groundbreaking study in which they identified a rare and transient subpopulation of totipotent-like cells within naïve mouse embryonic stem cell (mESC) cultures [1]. These cells spontaneously upregulated the expression of many genes exclusively expressed in 2-cell (2C) embryos. Moreover, these cells, which they called 2C-like cells (2CLCs), were able to give rise to both embryonic and extraembryonic tissues *in vivo*. The 2CLCs could be identified by expression of the retrotransposon MERVL, which in turn was used to generate a 2CLC reporter. However, only <1% of cells in naïve mESC culture were found to be in this 2C-like state at any given time. Nonetheless, this was the first report of totipotent-like cells in culture and inspired many studies aimed toward increasing the stability of totipotent-like cells. Later, the retrogene DUX was identified as the master regulator of entering the 2C-like state in mESCs. Using an inducible expression system, *Dux* was found to be both necessary and sufficient to convert naïve mESCs into the 2C-like state and was able to increase the proportion of 2CLCs from <1% to >70% [2]. In 2021, Shen et al. identified spliceosomal repression as a method to promote a pluripotent-to-totipotent transition using the splicing inhibitor pladienolide B, which allowed for the stabilization of totipotent blastomere-like cells [3]. Shortly after, two separate groups both generated novel mouse totipotent-like cells from mouse embryos and mESC cultures by modulating the activity of epigenetic enzymes [4, 5]. Despite the advances being made for stabilizing mouse totipotent-like cells, little progress had been made in establishing a human counterpart.

The identification of human totipotent-like cells in culture took an additional decade after the first identification of mouse 2CLCs. Totipotent-like human cells with human 8 cell (8C) embryo characteristics were first observed as a transient subpopulation during the late stages of human naïve PSC reprogramming [6]. Similarly, a small subpopulation of cells that transcriptionally resembled day 3 human embryonic cells were identified in human ­blastocyst-like structures generate from naïve hPSCs [7]. However, neither group attempted to isolate nor characterize the transient cells. Nonetheless, these cells were our first glimpse into human totipotency in culture.

**Figure 1.**
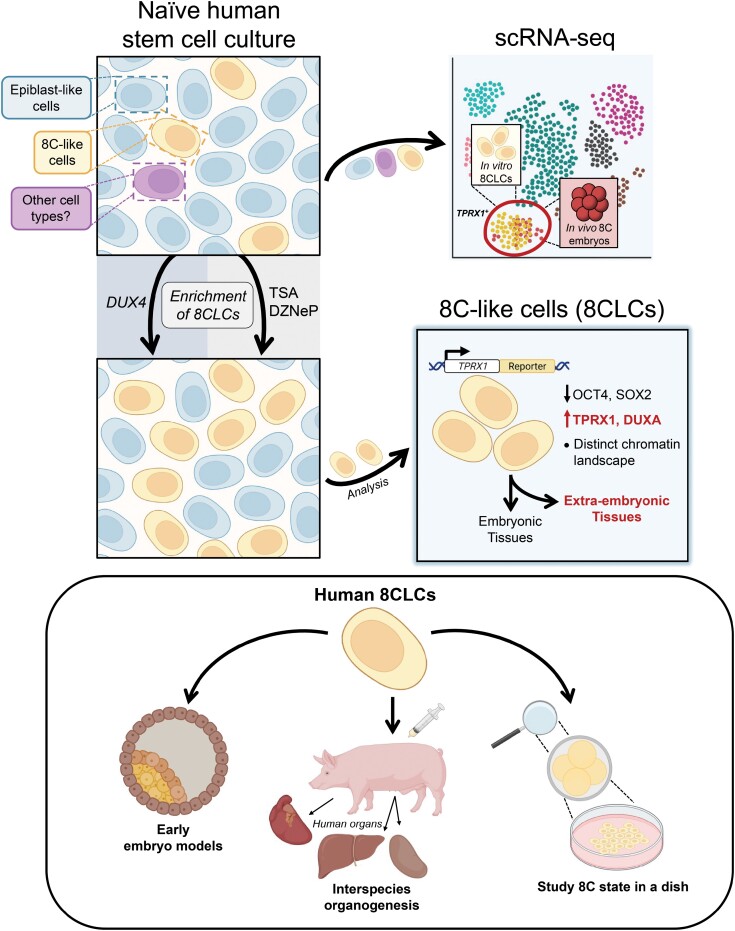
Capture of human 8CLCs in naïve pluripotent stem cell cultures. The heterogeneous gene expression patterns and plasticity of naïve PSCs allows subpopulations of 8CLCs to arise. 8CLCs cells were first identified by scRNA-seq and showed transcriptional similarity to *in vivo* human 8C embryos. The abundance of 8CLCs in culture can be improved by modulating the epigenetic and transcriptional state of naïve hPSCs via transgene expression (*DUX4*) or small molecule inhibitors [trichostatin A (TSA) and 3-deazaneplanocin A (DZneP)]. The transcriptional features of 8CLCs and 8C embryos identified through scRNA-seq allowed for the identification of the human 8C marker *TPRX1*, a key director of 8CLC gene regulatory networks. 8CLCs downregulate the expression of canonical pluripotency genes (e.g. OCT4 and SOX2) and upregulate totipotency-associated genes (e.g. TPRX1 and DUXA). They also display functional characteristics of totipotency as they can give rise to both embryonic and extraembryonic tissues *in vitro* and *in vivo*. 8CLCs hold great promise in advancing early embryo models, improving interspecies chimerism for interspecies organogenesis, and modeling human development in a dish. (Figure created using BioRender.com.)

Most recently, through scRNA-seq analysis, three separate groups identified and performed detailed characterization of cells that were transcriptionally similar to human 8C embryos, and thus named them 8C-like cells (8CLCs) (Fig. 1). Taubenschmid-Stowers et al. identified 8CLCs as a subpopulation within cultures of naïve hPSCs [8]. By comparing the expression profiles of human 8C embryos with *DUX4* overexpression cell lines, the authors generated a set of markers that were representative of human zygotic genome activation (ZGA), a process that occurs during the human 8C stage. Using this new dataset, 8CLCs were identified through scRNA-seq and represented ~1.5% of cells in naïve hPSC cultures. The 8CLCs could also be identified by high expression of *TPRX1*, a gene implicated in ZGA transcription, which allowed for the identification and analysis of 8CLCs within the naïve hPSC culture. In addition, similar to mouse 2CLCs, *DUX* overexpression increased the abundance of 8CLCs in culture by 20-fold. A separate study corroborates the finding of 8CLCs within naïve hPSC cultures [9]. Interestingly, this group also identified ­trophectoderm-like cells in the same cultures, further confirming the presence of multiple distinct cell types within naïve hPSC cultures.

At the same time, a third group established a naïve hPSC culture condition centered around the inhibition and promotion of histone methylation and acetylation, respectively, that promoted the expression of totipotency-associated genes (e.g. *TPRX1*, *ZSCAN4*, and *DUXA*) [10] (Fig. 1). Similar to the aforementioned studies, Mazid et al. found 8CLCs as a subpopulation within naïve hPSC cultures (~11% of cells). In addition, by generating a *TPRX1* expression reporter line, the authors were able to isolate populations of 8CLCs and revealed high-fidelity transcriptional and epigenetic features of human totipotency. These cells were also able to give rise to extraembryonic tissues both *in vitro* and *in vivo*. Perhaps most importantly, through ­scRNA-seq analysis, the authors defined a molecular roadmap of the ­pluripotent-to-totipotent conversion process, providing our first glimpse into the process of how human totipotent-like cells can be generated. This roadmap will undoubtedly expedite improvements to human totipotent-like cell cultures.

Despite these exciting advances, two predominate complications arise while assessing totipotency in human cells. First, unlike mouse cells that can be stringently assessed by performing *in vivo* integration assays in mouse embryos, ethical, and legal limitations prevent similar tests from being performed using human cells and human embryos. Second, similar to human totipotent-like cells, hPSCs themselves can generate extraembryonic tissues. These two confounding issues warrant the assessment of several criteria while evaluating the true potency of human totipotent cells, such as transcriptomic comparisons to bona fide embryos and *in vitro* differentiation assays, as performed by Mazid et al. However, as with human pluripotency, using the most stringent method to assess human totipotency (i.e. *in vivo* integration assays into human embryos) is not feasible and perhaps never will be.

All of the studies highlighted here leveraged the intrinsic cellular heterogeneity that is found within artificial cultures of naïve PSCs. Both mouse and human totipotent-like cells were discovered and isolated through a combination of transcriptomic analyses and the utilization of reporter genes. In these studies, the transcriptomic analyses allowed for the preliminary identification and characterization of totipotent-like cells, and the reporter gene [e.g. MERVL (mouse) and TPRX1 (human)] allowed for the isolation of totipotent-like cells from heterogeneous cultures of PSCs. The more recent studies on human totipotent-like cells utilized the high-resolution technique of scRNA-seq, which allows for cellular heterogeneity to be perceived on a cell-by-cell basis, and essentially characterizes every individual cell in a heterogeneous culture (Fig. 1). As exemplified by the research highlighted here, the combination of scRNA-seq with a plastic cell type of inherent heterogeneity (i.e. PSCs) is extremely powerful for the identification and eventual isolation of novel cell types with unique properties.

Together, these studies show that human totipotent-like cells can be identified within naïve hPSC cultures and that the culture conditions can be modulated to increase the stability and abundance of these cells. By embracing the artificial characteristics of naive hPSCs propagated *in vitro*, these studies revealed new avenues for the study and understanding of human totipotency. Perhaps there are additional unique cell types not yet discovered that can be identified within PSC cultures through similar strategies utilized in the research highlighted here. If there are, it would be interesting to see if the new cell types exhibit unique properties not yet captured *in vitro*, and whether these cells could be stabilized for further studies. Along the same lines, perhaps a similar strategy can be employed to generate artificial cell types with sought-after properties for biotechnological applications.

Customized stem cell lines with extraordinary properties designed for specific applications are steadily becoming a reality. The generation of stem cell lines that have totipotent-like properties is already a substantial leap forward in this direction, and these cells have immense potential for revolutionizing basic science, biotechnology, and translational medicine.
